# Discovery of SARS-CoV-2-E channel inhibitors as antiviral candidates

**DOI:** 10.1038/s41401-021-00732-2

**Published:** 2021-07-22

**Authors:** Yi Wang, Sui Fang, Yan Wu, Xi Cheng, Lei-ke Zhang, Xu-rui Shen, Shuang-qu Li, Jian-rong Xu, Wei-juan Shang, Zhao-bing Gao, Bing-qing Xia

**Affiliations:** 1 CAS Key Laboratory of Receptor Research, Stake Key Laboratory of Drug Research, Shanghai Institute of Materia Medica, Chinese Academy of Sciences, Shanghai, 201203 China; 2 University of Chinese Academy of Sciences, Beijing, 100049 China; 3 State Key Laboratory of Virology, Wuhan Institute of Virology, Center for Biosafety Mega-Science, Chinese Academy of Sciences, Wuhan, 430071 China; 4 Academy of Integrative Medicine, Shanghai University of Traditional Chinese Medicine, Shanghai, 201203 China; 5 Department of Pharmacology and Chemical Biology, Shanghai Jiao Tong University School of Medicine, Shanghai, 200025 China; 6 Zhongshan Institute of Drug Discovery, Institution for Drug Discovery Innovation, Chinese Academy of Science, Zhongshan, 528400 China

**Keywords:** SARS-CoV-2, envelope protein (2-E), cation channel, high-throughput screening (HTS), anti-virus

## Abstract

Lack of efficiency has been a major problem shared by all currently developed anti-SARS-CoV-2 therapies. Our previous study shows that SARS-CoV-2 structural envelope (2-E) protein forms a type of cation channel, and heterogeneously expression of 2-E channels causes host cell death. In this study we developed a cell-based high throughput screening (HTS) assay and used it to discover inhibitors against 2-E channels. Among 4376 compounds tested, 34 hits with cell protection activity were found. Followed by an anti-viral analysis, 15 compounds which could inhibit SARS-CoV-2 replication were identified. In electrophysiological experiments, three representatives showing inhibitory effect on 2-E channels were chosen for further characterization. Among them, proanthocyanidins directly bound to 2-E channel with binding affinity (*K*_D_) of 22.14 μM in surface plasmon resonance assay. Molecular modeling and docking analysis revealed that proanthocyanidins inserted into the pore of 2-E N-terminal vestibule acting as a channel blocker. Consistently, mutations of Glu 8 and Asn 15, two residues lining the proposed binding pocket, abolished the inhibitory effects of proanthocyanidins. The natural product proanthocyanidins are widely used as cosmetic, suggesting a potential of proanthocyanidins as disinfectant for external use. This study further demonstrates that 2-E channel is an effective antiviral drug target and provides a potential antiviral candidate against SARS-CoV-2.

## Introduction

As the SARS-CoV-2 pandemic unfolds across the globe, more than 100 million people have been diagnosed and 3 million deaths have been reported worldwide. Similar to the earlier SARS and MERS beta coronaviruses (SARS-CoV, MERS-CoV), SARS-CoV-2 primarily infects alveolar epithelial cells of the lung and contributes to multiple organs failure and even death [1, 2]. Though vaccines and neutralizing antibodies are the most promising treatments currently [3, 4], looking for small molecule drugs is a long-term way for therapy. Among the 2036 clinical trials till October 2020, 73% of cases are associated with small molecule drugs development [5]. There are two categories for potential anti-coronavirus therapies. One targets SARS-CoV-2 itself and the other acts on the host immune system or host cell [6, 7]. As a novel coronavirus, SARS-CoV-2 contains four structural proteins, nonstructural proteins, and some accessory proteins [8–11]. To date, targeting viral itself to discover potential drugs has obtained certain achievements in the progress of fighting against coronavirus. For example, remdesivir, an RNA-dependent RNA polymerase inhibitor, blocks the replication of virus [12]. Lopinavir/ritonavir and GRL0617, inhibitors of viral protease, inhibit virus maturation [13, 14]. Similarly, baicalin and baicalein, the main protein (M^pro^) inhibitors, also exhibit antiviral activity [15, 16]. In addition, some traditional Chinese medicines such as Lianhuaqingwen, were reported to reduce viral replication and improve the clinical symptoms of COVID-19 through its high inhibitory effect on human angiotensin-converting enzyme 2 (hACE2) [17–19]. On the other side, drugs that target host cells, such as dexamethasone, hydroxychloroquine, and chloroquine have been used for the treatment of COVID-19 perhaps through enhancing the innate immune system and attenuating the inflammatory response [20–22]. Plitidepsin, a eukaryotic elongation factor 1A (eEF1A) inhibitor, exhibited potent preclinical efficiency against SARS-CoV-2 [23]. Though many potential drugs partly have anti-coronavirus effects in animal infection models and have been evaluated in ongoing clinical trials, there is no specific drug for COVID-19 currently. Partial broad-spectrum antiviral drugs that have been used clinically were withdrawn for various reasons or only used in emergency situations. For instance, hydroxychloroquine has been withdrawn by the US Food and Drug Administration due to its strong side effects, such as heart rhythm disturbances [24]. Remdesivir shortens the time to recovery in mild patients whereas has no effect in severe patients [25]. Dexamethasone only modestly reduces mortality of patients who are receiving respiratory support [26]. Thus, discovery of effective targets and specific drugs with low side effects is still extremely urgent.

Previously, we have demonstrated that SARS-CoV-2 envelope protein (2-E) forms a type of cation channel and leads to host cell death [27]. We also found that the 2-E channel inhibitors are able to protect host cells from damage and exhibit anti-SARS-CoV-2 activity, suggesting a possibility of identifying antiviral hits through evaluating cell-protective efficiency of compounds. Accordingly, we developed and carried out a high throughput screening (HTS) assay to find antiviral candidates targeting 2-E protein.

## Materials and methods

### Plasmid and compound libraries

The 2-E sequence was synthesized by the Beijing Genomics Institute (BGI, China). Vector pET28a and pcDNA5 were used for protein purification and cell transfection, respectively. Point mutations were generated using site-directed mutagenesis and confirmed by sequencing. Totally 4376 small molecules from multiple libraries were screened, including the US compound library (~1200 compounds), the LJ compound library (~800 compounds), the Chinese National Compound Library (~2300 compounds). Veliparib (HY-10129, CAS No. 912444-00-9), wortmannin (HY-10197, CAS No. 19545-26-7), proanthocyanidins (HY-N0794, CAS No. 4852-22-6) were purchased from MedChemExpress (MCE, NJ, USA).

### Cell culture

Vero E6 cells were purchased from National Collection of Authenticate Cell Cultures (China). Vero E6 cells were grown in 90% DMEM basal medium (11054001, Gibco, NY, USA) supplemented with 10% fetal bovine serum (10100, Gibco, NY, USA) and 100 units/mL penicillin/streptomycin (15070063, Gibco, NY, USA). Cells were grown at 37 °C, 5% CO_2_ incubator, and passaged approximately every 2 days when confluency up to 80%–90%.

### High throughput screening assay

For the first round of primary screening, Vero E6 cells were seeded in 96-well plates with 4,000 cells per well overnight. In the next day, 10 μM compounds were pre-incubated with Vero E6 cells for 6 h with duplicate wells, then we transfected Vero E6 cells with 400 ng/well 2-E plasmids using Lipofectamine 3000 Transfection Reagent (L3000015, Thermo Fisher, MA, USA). After 24 h (the third day), we tested cell viability by CCK-8 kit (40203ES60, Yeasen, China) according to the manufacturer’s instructions. For the second round, we transfected Vero E6 cells first, after 6 h added 10 μM compounds with six repeats, and tested cell viability by CCK-8 kit 24 h later. For cytotoxicity assay, only 10 μM compounds were incubated with Vero E6 cells for 24 h. Absorbance analysis were performed with Thermo Scientific Microplate Reader at 450 nm according to the manufacturer’s instructions (Thermo Fisher, MA, USA). Cell Protection% = (*A*_control_ – *A*_drug_)/(*A*_control_ – *A*_model_) %. *A*_control_, *A*_drug_, and *A*_model_ represented absorbance value of three groups respectively at 450 nm via CCK-8 assay. The control group was Vero E6 cells transfected with 400 ng/well pcDNA5 vector plasmids alone. The model group was Vero E6 cells transfected with 400 ng/well 2-E plasmids alone. The drug group means the compounds treated Vero E6 cells with 400 ng/well 2-E plasmids transfection.

### Single-channel electrophysiological recording

The 2-E protein was expressed in *E. coli* BL21/DE3 pLysS cells and purified with Ni-NTA column, referred to our previous description [27]. Then purified 2-E protein was incorporated into lipid bilayers to examine the channel inhibition of compounds. All the lipids were bought from Avanti (850356P, 850408P, Avanti Polar Lipids, AL, USA). The membrane lipids consist of phosphatidylcholines/phosphatidylserine = 3/2. The buffer contained 500 mM KCl in *cis*/50 mM KCl in *trans*, all solutions were buffered by 5 mM HEPES, pH = 6.35. Detailedly, proteins were added to *cis* side. Agitation and electrochemical gradients induced 2-E to combine with lipid membrane. Membrane currents were recorded under voltage-clamp mode using a Warnner bilayer clamp amplifier BC-535 (Warner Instruments, MA, USA), filtered at 1–2 kHz. The recording frequency was 10 kHz. The currents were digitized using pClamp 10.2 software (Molecular Devices, CA, USA). The single-channel conductance was determined by fitting to Gaussian functions (bin width = 0.25 pA) and the open time was determined by fitting to single or bi-exponential equations. Open times less than 0.5–1.5 ms were ignored. Channel inhibition% = (1 − *P*_o, drug_/*P*_o, control_)%. *P*_o, control_ and *P*_o, drug_ represented 2-E channel open probability before and after drug treatment. *P*_o_, the probability of channel being open, for the currents from a single channel was defined from the amplitude histogram as the ratio of the area from open channels to the total area.

### Antiviral efficiency test for compounds

Vero E6 cells were seeded in 48-well plate with 4 × 10^4^ cells per well overnight. The next day, compounds diluted as 10 μM were incubated with the cells for 1 h at 37 °C. Then SARS-CoV-2 (isolatenCoV-2019BetaCoV/Wuhan/WIV04/2019) (about 400 PFU in 10 μL DMEM, MOI = 0.01) was added. Twenty-four hours later, cell supernatants were taken for viral copies detection as usual. Viral RNA was isolated with MiniBEST Viral RNA/DNA Extraction Kit (9766 A, Takara, Japan) as the instruction described, and transcribed with PrimeScript^TM^ RT reagent Kit with gDNA Eraser (RR047B, Takara, Japan). Viral copies were quantified from viral cDNA by RT-PCR (Takara TB Green^®^ Premix Ex Taq™ II, Takara, Japan) with a standard curve method on ABI 7500 (Applied Biosystems 7500, CA, USA). The sequences of primers targeting SARS-CoV-2 S gene were as follows: F: 5′-CAATGGTTTAACAGGCACAGG-3′; R: 5′-CTCAAGTGTCTGTGGATCACG-3′. Antiviral efficiency (Inhibition %) = (1− *V*_drug_/*V*_control_) %. *V*, represented viral copies. The control group and drug group were defined as 1% DMSO or compounds were incubated with Vero E6 cells for 1 h at 37 °C, then Vero E6 cells were infected with SARS-CoV-2 (about 400 PFU in 10 μL DMEM, MOI = 0.01). After 24 h, cell supernatants were taken for viral copies via qRT-PCR.

### Molecular docking

The solid-state NMR structure of SARS-CoV-2 envelope protein (PDB code: 7K3G) [28] was used as the docking receptor. A ligand was docked to the receptor using Schrodinger Glide software in SP mode with extended sampling. Searches were performed using Lamarckian Genetic Algorithm with default settings. A ligand was initially placed in the center of the binding site. Its center of mass was constrained to move within 1 nm diameter sphere, where it was allowed moving freely during the docking process. The docking model with the lowest binding energy was selected for analysis.

### SPR assay

Biacore T200 instruments (GE Healthcare, UK) were used to evaluate the binding affinity of compounds to 2-E protein via SPR, as previously described [29]. Briefly, 2-E protein was immobilized on the surface of CM5 chip by using amine-coupling approach at a flow rate of 10 μL/min in 10 mM sodium acetate buffer (pH 4.5). The sensor surface was activated with a 7 min injection of the mixture of 50 mM N-hydroxysuccinimide (NHS) and 200 mM 1-ethyl-3-(3-dimethylaminopropyl) carbodiimide. Then 50 μg/mL of Tau protein was injected to reach the target level of around 1400 RU and the surface was blocked with 1 M ethanolamine, pH 8.5. Series concentrations (typically 12.5, 25, 50, 100, 200 μM) of compounds were injected into the flow system and analyzed for 90 s, and the dissociation was 120 s. All binding analysis was performed in phosphate-buffered saline with 0.05% (*v*/*v*) Tween-20 and 1% DMSO, pH 7.4, at 25 °C. Prior to analysis, double reference subtractions were made to eliminate bulk refractive index changes, injection noise, and data drift. The binding affinity was determined by fitting to a Langmuir 1:1 binding model within the Biacore Evaluation software (GE Healthcare, UK).

### Statistical analysis

The data are presented as the mean ± SEM. The dose-effect curve was fitted using the Hill equation.

## Results

### Primary screening identified hits with protection activity against 2-E-induced cell death

**Fig. 1:**
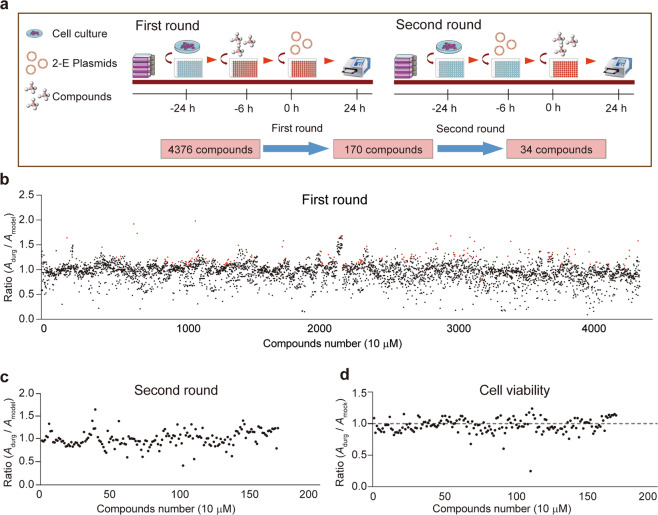
Primary screening identified hits with protection activity against 2-E-induced cell death. **a** Schematic of cell based high throughput screening assay. **b** Overview of first round result. All compounds (4376) and their corresponding activity are represented by circle. The cell viability ratio of *A*_drug_/*A*_model_ displayed along the vertical axis. Top five to eight compounds with a ratio greater than 1 in each plate were considered as effective inhibitors and were displayed in red circle. Data represent the mean of the duplicate results. **c** Cellular protective ratio of secondary round screening (170 hits). Data represent the mean of the six repeats. *A*_model_, the absorbance of 2-E transfected cells with DMSO treatment group. *A*_drug_, the absorbance of 2-E transfected cells with 10 μM drug treatment group. **d** Cell viability ratio of 170 positive compounds. Data represent the mean of the six repeats. *A*_drug_, the absorbance of 10 μM drug treatment group. *A*_mock_, the absorbance of DMSO treatment group.

We established a HTS assay by measuring protection activity of compounds against 2-E induced cell death. In the first round of the primary screening, Vero E6 cells were pre-incubated with 10 μM compounds and then transfected with 2-E plasmids. After 24 h, we assessed cell viability by measuring the change of absorbance (*A*) in the presence or absence of compounds (Fig. 1a). We defined the cell viability ratio of *A*_drug_/*A*_model_ > 1 as an effective compound. After the first round, 170 (3.9%) compounds were considered as pre-hits among 4376 tested compounds (Fig. 1b, red circles). Considering expression inhomogeneous of 2-E due to the variation of transient transfection efficiency among different plates, top five to eight compounds from each plate were selected for re-evaluation. Instead of pre-incubation, in the second round of the primary screening, 10 μM compounds were applied 6 h after transient transfection (Fig. 1c). Only those compounds showing protective activity in both two rounds were considered as effective. In addition, those compounds that promoting cell proliferation were further removed from the hit list (Fig. 1d). Finally, 34 hits (0.8%) with protection activity against 2-E induced cell death were identified (Table 1).

**Table 1:** Summary of cell protection, channel inhibition, and antiviral activity of 34 compounds.

Compound name	Channel inhibition (100 μM)	Cellular protection (10 μM)	Antiviral activity (inhibition%, 10 μM)
2-Deoxy-D-glucose	**-**	1.34	<20
Talazoparib	**-**	1.43	20–40
Veliparib	+	1.34	>40
Carsalame	**-**	1.13	>40
Gossypol-acetic acid	**-**	1.41	>40
Salvianolic acid B	**-**	1.39	<20
GW4869	**-**	1.29	20–40
Wortmannin	+	1.30	>40
Demethyl	**-**	1.18	>40
COH29	**-**	1.28	>40
IPA-3	**-**	1.27	<20
BMS-626529	**-**	1.21	<20
AT101	**-**	1.36	20–40
Quercetin	**-**	1.26	20–40
Kartogenin	**-**	1.27	20–40
Edoxaban	**-**	1.35	<20
Rifamycin sodium	+	1.21	<20
Midecamycin	+	1.20	<20
Dihydroartemisinin	+	1.37	<20
Proanthocyanidins	+	1.92	20–40
Artemisinin	+	1.22	20–40
Artesunate	**-**	1.49	<20
Phenindione	**-**	1.20	<20
Dihydroergotamine mesylate	+	1.14	<20
Evans blue	**-**	1.58	<20
Streptomycin sulfate	**-**	1.18	<20
Nafronyl oxalate	+	1.24	<20
Dimethyl fumarate	+	1.14	<20
Valdecoxib	+	1.37	<20
Chloropyramine hydrochloride	**-**	1.36	>40
ABT-199 (GDC-0199)	**-**	1.22	20-40
Sapogenins Glycosides	**-**	1.15	<20
Carbamazepine	**-**	1.17	<20
Oxybutynin	**-**	1.47	<20

“+” means the inhibition of 2-E channels; “-” means no inhibition of 2-E channels.

### Secondary screening verified hits with antiviral activity and 2-E channel inhibition ability

**Fig. 2:**
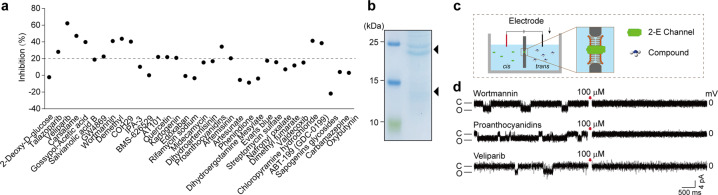
Characters of 34 hits on anti-viral activity and 2-E channel inhibition ability. **a** Anti-SARS-CoV-2 efficiency of 34 hits at 10 μM. Vero E6 cells were pre-treated with compounds for 1 h, and then infected with SARS-CoV-2(MOI = 0.01). Viral copies were detected through qRT-PCR. **b** Purification of full-length 2-E protein with Ni-NTA affinity chromatography. 15% SDS-PAGE gel with coomassie blue staining. Triangle, 2-E proteins [27]. **c** Schematic of planar lipid bilayer system. **d** Representative single-channel traces after 2-E exposed to the indicated compounds at 100 μM. Once ion channel conductance was detected, compounds were added to the *trans* side while stirring to facilitate binding of the compound to the channel. The black arrow indicates the application of compounds (*n* ≥ 3). “C” means channel close; “O” means channel open.

Thirty-four hits with antiviral efficiency against SARS-CoV-2 were evaluated in the secondary screening. Vero E6 cells were pretreated with 10 μM compound for 1 h, then infected with virus (MOI = 0.01). Dimethyl sulfoxide (DMSO, 1%) was used as control. Inhibition efficacy was evaluated by measuring viral copy numbers in the cell supernatant via qRT-PCR. Defined inhibition > 20% as the standard, 15 compounds significantly suppressed SARS-CoV-2 replication (Fig. 2a). Subsequently, the inhibitory activity of these compounds on 2-E channels was further examined using the planar lipid bilayer (BLM) system. We defined the protein added side as *cis* side and the opposite as *trans* side (Fig. 2b,c). After ion channel conductance was detected, 100 μM compounds were added to the *trans* side while stirring to facilitate binding of the compound to the channel. Three of the antiviral compounds, wortmannin, proanthocyanidins, and veliparib, were found to effectively inhibit 2-E channel currents (Fig. 2d). Wortmannin is currently used as a pharmacological tool compound. Proanthocyanidins are a class of natural products. Being an antitumor drug, veliparib is currently in phase III clinical trial.

### Validation of proanthocyanidins as an antiviral candidate targeting 2-E channels

**Fig. 3:**
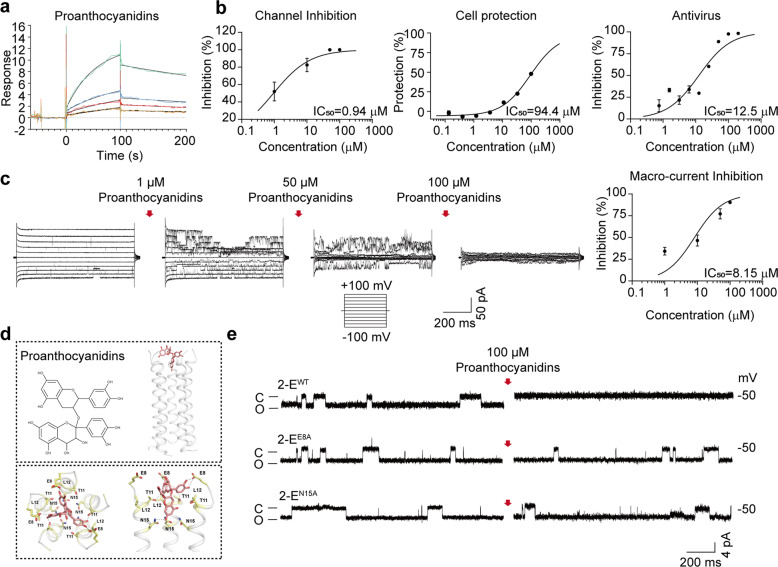
The interaction mechanism of proanthocyanidins with the 2-E channel. **a** Binding ability of proanthocyanidins, wortmannin, and veliparib to 2-E channel via SPR. **b** IC_50_ of proanthocyanidins on cell protection, channel inhibition, and antivirus. **c** Proanthocyanidins inhibited 2-E-induced macro-currents. Left, proanthocyanidins dose-dependently inhibited 2-E-induced macro-current. Right, IC_50_ of proanthocyanidins on macro-currents inhibition (*n* = 3). **d** A representative docking pose of proanthocyanidins. The protein was shown in cartoon depict. The compound and key residues are shown as sticks. The putative hydrogen bonds are shown as dash lines. **e** Representative single-channel traces after the indicated 2-E mutant channels exposed to 100 μM proanthocyanidins at −50 mV. Once ion channel conductance was detected, compounds were added to the *trans* side while stirring to facilitate binding of the compound to the channel. The red arrow indicates the application of compounds (*n* ≥ 3). “C” means channel close; “O” means channel open.

To explore whether the channel inhibitory activity of the three compounds was due to direct binding, the SPR assay was performed. Dose-dependent binding of proanthocyanidins with 2-E proteins was observed (Fig. 3a). The binding interaction exhibited a fast association rate and a slow dissociation rate, indicating that proanthocyanidins presented relatively strong affinity for 2-E proteins. The response units at equilibrium were plotted against the proanthocyanidins concentration, and the dissociation constant (*K*_D_) was calculated by fitting these data to fit curves through non-linear regression analysis. The results showed that proanthocyanidins bound to 2-E with a *K*_D_ value of 22.14 μM (Fig. 3a). In contrast, at equivalent molar ratios, wortmannin and veliparib exhibited weak or undetectable binding with 2-E proteins (Supplementary Fig. S1).

Then, proanthocyanidins were chosen for further evaluation. We found that proanthocyanidins dose-dependently suppressed 2-E induced Vero E6 cell death, 2-E channel currents, and the virus replication (Fig. 3b). The IC_50_ of proanthocyanidins were 94.4 μM, 0.94 μM, 12.5 μM for cell protection, channel inhibition, antiviral activity, respectively. Noticeably, under tested concentrations, no significant cytotoxicity was detected (CC_50_ > 500 μM) (Supplementary Fig. S1c). In addition, we also evaluated proanthocyanidins effects on 2-E channel induced macroscopic currents under symmetric potassium solutions via BLM. After 2-E channel incorporated in lipids, we added different concentrations of proanthocyanidins in *trans* side. The macroscopic currents were inhibited depending on the proanthocyanidins concentrations (Fig. 3c) with an IC_50_ = 8.15 μM. Molecular docking of proanthocyanidins against the solid-state NMR structure of 2-E channel was carried to investigate how proanthocyanidins interact with the channels [28]. The docking model showed that the proanthocyanidins bind to the N-terminal vestibule of the 2-E protein, suggesting the compound may inhibit cation conduction by steric occlusion of the pore. In the N-terminal vestibule of E protein, four different residues (E8, T11, L12, and N15) from different subunits constitute a binding pocket of the proanthocyanidins (Fig. 3d). The docking poses of the proanthocyanidins showed that their chromane-5,7-diol group inserts into the pore of the E protein and forms putative hydrogen bonds with a polar residue N15. Linked to the chromane-5,7-diol group of the compound, the benzene-1,2-diol group attaches to the edge of the binding pocket consisting of T11 and L12 and forms putative hydrogen bonds with a negatively charged residue E8 (Fig. 3d). The rest part of the compound (i.e., 2-(3,4-dihydroxyphenyl) chromane-3,4,5,7-tetraol group) lies on the mouth of the pore and forms multiple putative hydrogen bonds with the charged residues E8 from different subunits. To validate the proposed binding site of proanthocyanidins, we designed single-point mutations engaging four residues mentioned above (2-E^E8A^, 2-E^T11A^, 2-E^L12A^ and 2-E^N15A^) and tested inhibitory capability of proanthocyanidins on these mutants. 2-E^E8A^ and 2-E^N15A^ mutations abolished the inhibitory effects of proanthocyanidins on the 2-E channel (Fig. 3e, Supplementary Figs. S2, S3), highly consistent with our molecular docking models. Taken together, these results validated proanthocyanidins as an antiviral candidate against SARS-CoV-2 targeting 2-E channels.

## Discussion

The absence of specific drugs against SRAS-CoV-2 has led humans to suffer the virus and major loss of economy for a long time [30, 31]. Drug discovery usually takes a long time and undergoes appropriate verification to ensure the safety and effectiveness of the drug. Under this circumstance, drug-repurposing is one of the attractive ways to find drugs for SARS-CoV-2 treatment. Our previous work found 2-E channel as an independent virulence factor not only causes the death of host cells but also participates in the life cycle of SARS-CoV-2 [27]. Based on the preliminary work, the current study revealed three major findings. First, we established a high throughput screening assay targeting cell lethal effect of 2-E channel; second, we discovered a 2-E channel inhibitor showing antiviral efficiency; third, our in vitro binding and simulation data highlighted proanthocyanidins binding pocket.

To date, HTS strategies have been widely used in drug discovery and three general HTS approaches were reported to discover potential anti-coronavirus treatment options [11, 32–35]. First, directly screen existing broad-spectrum antiviral drugs that have been used to treat other viruses. Second, screen and repurpose chemical libraries or database that contain known drug effects and known information, such as signal pathways, physiological and/or immune responses. Third, de novo discover new drugs based on the genome and biophysical understanding of the coronavirus [32, 36, 37]. These schemes were effective to find candidate molecules but still existed an imperfection. For instance, screening of existing broad-spectrum antiviral drugs is not specific and may bring many side effects. The inhibitors targeting host cell hACE2, an important regulator of systemic blood pressure and cardiovascular disease, might cause heart and blood toxicity [38]. Besides, the redevelopment of new drugs not only requires huge manpower, material resources, financial resources, but also spends a long period. The HTS by measuring the influence of compounds on 2-E-induced cell death exhibits advantages. First, 2-E channel is a target on virus itself which may avoid the potential side effects mediated by host cell targets. Second, CCK-8 based method is easy to carry out and permits us obtaining a large amount of effective data quickly. The current pilot screening demonstrated the cell-based HTS is a promising strategy to discover antiviral candidates.

Proanthocyanidins are known as condensed tannins that are widely identified in various plants [39]. It has been endorsed to possess significant antioxidant capacities and owned various pharmacological properties. On one hand, proanthocyanidins have been developed as a skincare product with anti-wrinkle, sunscreen, and whitening abilities [40, 41]. On the other hand, proanthocyanidins has therapeutic effects on a variety of diseases, such as cardiovascular diseases, neurological diseases and tumors [42–46]. It is worth to note, proanthocyanidins could inhibit the growth of liver cancer cells through specifically block Kv10.1 channel [47]. Very recently, virtual screening found proanthocyanidins could inhibit main protease (M^pro^) activity of SARS-CoV-2, whereas lacked of antiviral activity evidence. In addition, many researchers reported proanthocyanidins antiviral effects including rotavirus, herpes simplex virus, coxsackie B3 virus, and hepatitis C virus [48–51]. Here, we first reported proanthocyanidins acting as a 2-E channel blocker weakens SARS-CoV-2 replication in vitro which works at a micromolar concentration of 12.5 μM. To decipher the working of the drug and mechanism of inhibition at the molecular level, we investigated protein-drug interactions via SPR and docking. The SPR finding suggests that the good affinity of proanthocyanidins with 2-E channel. Molecular modeling studies give solid support to the fact that proanthocyanidins bind to pore pocket of 2-E channel N-terminal. In addition, our molecular modeling and electrophysiological data demonstrated that proanthocyanidins interact with E8 and N15 residues, which are critical pore-facing position amino acids [28].

The structural envelope (E) protein shares striking functional similarities in different coronaviruses, including SARS-CoV, MERS-CoV [52]. In addition to the essential roles of 2-E channel we identified, it has been found that deletion of E channel results in attenuating SARS-CoV pathogenesis [53–55]. Beyond that, E channel was also found to participate in MERS-CoV assembling, virion release, and pathogenesis [56, 57]. Thus, the small molecules targeting SARS-CoV-2 envelope protein could be potential broad-spectrum anti-coronavirus drugs. The limitation of our study is lacked in vivo validation, which is mainly due to the scarcity of P3 Labs and we long for sharing our results immediately. In brief, we established an efficient screening system targeting 2-E channel and identified potential therapeutic candidates against SARS-CoV-2.

## Supplementary information

Supplementary Fig. S1

Supplementary Fig. S2

Supplementary Fig. S3
