# Supplementary figures and images for: Discovery of SARS-CoV-2-E channel inhibitors as antiviral candidates
Source: Acta Pharmacol Sin. 2021 Jul 22;43(4):781–7. doi: 10.1038/s41401-021-00732-2 (PMC8295639; doi:10.1038/s41401-021-00732-2)

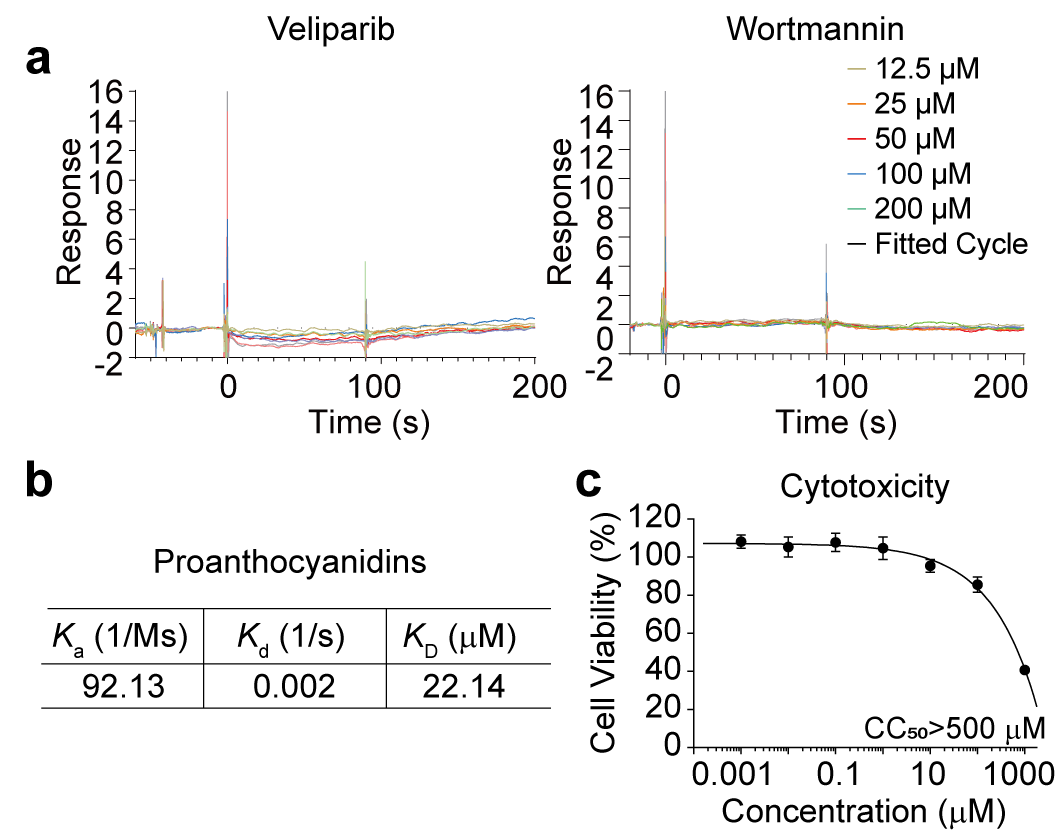

Supplement: Supplementary file 1 — Supplementary Fig. S1 [file 41401_2021_732_MOESM1_ESM.tif]

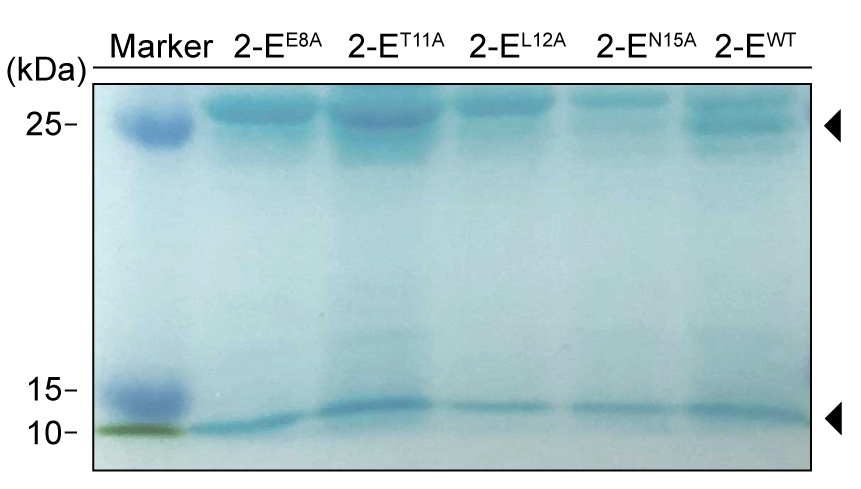

Supplement: Supplementary file 2 — Supplementary Fig. S2 [file 41401_2021_732_MOESM2_ESM.tif]

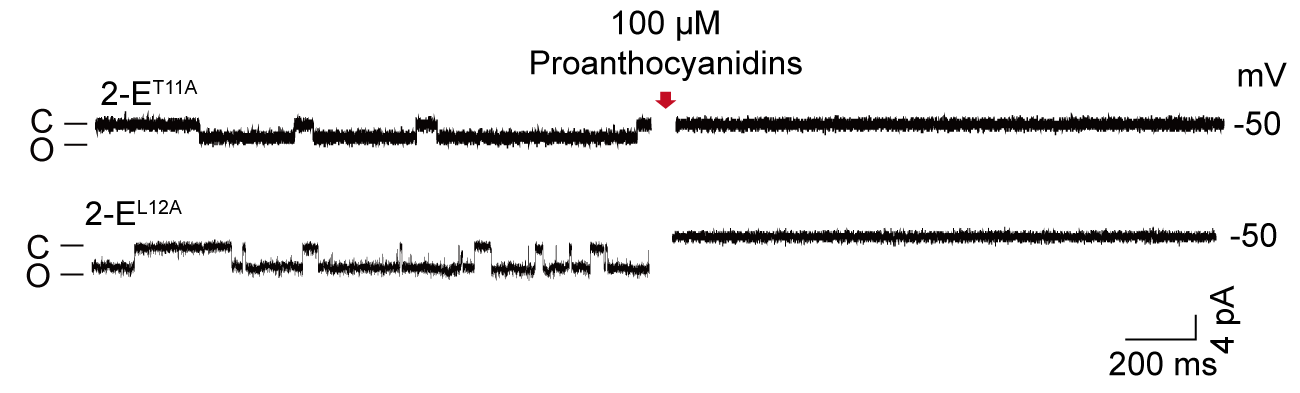

Supplement: Supplementary file 3 — Supplementary Fig. S3 [file 41401_2021_732_MOESM3_ESM.tif]
